# Development of the Ajinomoto Group Nutrient Profiling System for Japanese Meals

**DOI:** 10.3389/fnut.2025.1568181

**Published:** 2025-05-21

**Authors:** Hiroko Jinzu, Keishiro Arima, Hiroaki Kobayashi, Shunji Sakai, Sachi Nii, Yuki Nakayama, Yuki Okabe, Chie Furuta

**Affiliations:** Institute of Food Sciences and Technologies, Ajinomoto Co., Inc., Kawasaki, Japan

**Keywords:** nutrient profiling system, dietary recommendations, nutritional value, food culture, Japanese meals

## Abstract

**Introduction:**

Nutrient profiling (NP) is a method used to classify or score foods based on their nutritional content and impact on human health. The Ajinomoto Group Nutrient Profiling System (ANPS) was previously developed to evaluate the nutritional value of cooked dishes consumed in Japan. This study aimed to develop a novel NP model, named the Ajinomoto Group Nutrient Profiling System for Japanese Meals (ANPS-Meal), to evaluate meals.

**Methods:**

The ANPS-Meal evaluates meals using essential components based on public health concerns specific to Japan. The scoring algorithm includes protein and vegetables for encouraged intake and saturated fatty acids and sodium for limited intake. The convergent validity of this model was verified by comparison with the metric Healthy Eating Index-2015 (mHEI-2015) and the Nutrient-Rich Food Index 9.3 (NRF9.3).

**Results:**

A total of 1,816 meals commonly consumed in Japan were evaluated using the ANPS-Meal. The average ANPS-Meal score was 73.7 (standard deviation = 15.5), with a median of 75 (interquartile range = 62.5–85) and a range of 27.5–100. High-scoring meals featured low sodium and high vegetables. The higher quartiles of the ANPS-Meal were associated with higher carbohydrate, dietary fiber, potassium, calcium, magnesium, iron, and vitamins A, D, and C, as well as lower fat content. Spearman’s correlation coefficients were *r* = 0.59 for the mHEI-2015 and *r* = 0.40 for the NRF9.3.

**Discussion:**

The newly developed ANPS-Meal can be used for evaluating the overall nutritional value of a wide variety of meals based on four components: protein, vegetables, saturated fatty acids, and sodium. This model provides a comprehensive tool for assessing meal quality in alignment with public health objectives specific to Japan.

## 1 Introduction

Nutrient profiling (NP) is the science of classifying and/or ranking foods based on their nutritional composition (1). In parts of the EU, nutrient profiling has been promoted to foster healthy eating habits and prevent disease (2–4). NP is commonly used to evaluate the nutritional value of foods and regulate health claims, front-of-pack (FoP) food labeling, food classification for subsidies and taxation, pricing, and advertising of unhealthy foods and drinks aimed at children (5–7).

NP is implemented using the NP model (NPM), an algorithm designed to classify or score foods based on the nutritional contents and impact on human health for specific purposes, generating scores or rankings that reflect how healthy foods are (8). For example, FoP labels provide intuitive, easy-to-understand information and may serve as an effective means of addressing public health concerns. FoP labels using the Health Star Rating introduced in Australia and New Zealand resulted in positive or permanent behavioral changes in individuals (9). Furthermore, Nutri-Score labeling, adopted in several European countries, has become widely known and has had a positive impact on self-reported purchasing behavior (10). Moreover, there is a relationship between the Food Standards Agency nutrient profiling system (FSAm-NPS), used to derive food items’ Nutri-Score, and mortality from all causes, cancer, and diseases of the circulatory, respiratory, and digestive systems (11). These findings suggest that providing information and communicating through labels can be useful tools to help consumers choose healthier items and foods, thereby leading to improvements in public health.

Most NPMs evaluate the nutritional components of individual foods. Consequently, they have become widely used in cultural contexts where processed foods are frequently consumed. However, in Southeast Asian countries, the frequency of consumption of unprocessed foods is high (12, 13); thus, the development of an NPM applicable to cooked dishes is warranted. To address this issue, the Ajinomoto Group Nutrient Profiling System (ANPS) for Dish (ANPS-Dish) was proposed to evaluate the overall nutritional value of cooked dishes (14). The ANPS-Dish is an NPM developed specifically for the Japanese, whose burden of noncommunicable diseases is increasing due to aging and lifestyle changes, in accordance with World Health Organization (WHO)’s recommendation (15). In Japan, excessive sodium intake is a significant concern, with previous studies reporting that over 88% of participants consumed more sodium than the tentative dietary goals for preventing lifestyle-related diseases (16). For these reasons, the ANPS-Dish is highly sensitive to the scoring algorithm for sodium. Furthermore, the algorithm was designed to use a minimal number of evaluation factors, emphasizing practicality and facilitating its application as a tool for developing proprietary seasonings and nutritional dishes as well as for making informed recommendations to consumers.

However, considering that the Japanese government recommends a meal pattern consisting of a staple food, a main dish, and a side dish for well-balanced meals (17). While also noting the synergistic and interactive effects of individual foods and nutrients that constitute a meal, the NPM, which can evaluate the nutritional value of a single meal, may also be useful (18, 19). Previous studies have developed the Breakfast Quality Index (20) and Breakfast Score (21) to evaluate the nutritional quality of the breakfast consumed by children and adolescents, as well as the Meal Index of Dietary Quality (22) and Healthy Meal Quality (23) to assess the quality of lunch; however, all of these instruments focus on assessing the quality of specific meals in specific meal occasions, while the number of NPMs that can assess the nutritional value of diverse single meals in any meal occasions is limited internationally. Therefore, this study aimed to develop an NPM that can evaluate the nutritional value of all meal types predominantly consumed in Japan.

## 2 Materials and methods

### 2.1 Scope and principles of the ANPS-Meal

This study aimed to develop the ANPS-Meal, a measure specifically designed to evaluate the nutritional quality of meals consumed in Japan. Eating patterns in Japan are becoming increasingly complex and diverse due to not only the traditional Japanese diet but also the Westernization of the Japanese diet (24). However, for the ANPS-Meal to be easily applicable to dietary recommendations for individual dietary occasions, it should be designed to allow the evaluation of diverse meals using only essential components based on public health concerns specific to the Japanese population. Consequently, we devised the ANPS-Meal, a region-specific NPM for meals in the Japanese population, based on the ANPS-Dish (14) through the following steps: (1) selection of components and daily values, (2) development of a scoring algorithm, and (3) validation of the ANPS-Meal.

### 2.2 Scoring algorithm of the ANPS-Meal

We selected protein and vegetables as the encouraged intake components and saturated fatty acids (SFAs) and sodium as the limited intake components in the ANPS-Meal. Further details are provided in the Results section. The component points are calculated based on the serving size. The points for the encouraged intake components (proteins and vegetables) were set between 10 and 0 on a one-point scale, decreasing by 10% relative to the target values. The maximum points for protein and vegetables indicated adequate protein and vegetable content. The points for SFAs, which is the limited intake component, were also set between 10 and 0 on a one-point scale, increasing by 10% with respect to the target values. For sodium, the points ranged between 10 and 0 on a 0.5-point scale. The maximum points of SFAs and sodium indicate adequate contents of SFAs and sodium, indicating a lower content. The total score of the ANPS-Meal was calculated as the sum of the points and multiplied by 2.5 to convert it to a 100-point scale.

### 2.3 Data of meals in the Japanese diet

We selected four meal groups—nutritionally recommended meals, randomly generated meals, restaurant meals, and bento-box meals—that are commonly consumed by Japanese people and reflect diverse eating occasions.

#### 2.3.1 Nutritionally recommended meals

The meals were selected from commercially available recipe books supervised by hospital institutions, excluding those specifically designed for patients with significant dietary restrictions. From these recipe books (25–32), 310 meals, each comprising a combination of staple foods, main dishes, and side dishes, some intended specifically for breakfast, lunch and dinner were analyzed, excluding recipes of single-item dishes. Furthermore, the energy content and nutrient values of the meals were calculated by five researchers, including two registered dietitians, based on the ingredients and weight values listed in recipe books using the Standard Tables of Food Composition in Japan (8th Revised Edition).

#### 2.3.2 Randomly generated meals

We generated 1,000 meals by randomly combining dishes from Excel Eiyo-kun ver. 8 (Kenpakusha, Tokyo, Japan) using Python 3.10.0. In Japan, these dishes are commonly consumed at home. We created 250 meals for each of the following four patterns of category combinations: (1) staple food (white rice), main dish, side dish, and soup; (2) staple food (white rice), main dish, and two kinds of side dishes; (3) staple food with soup (≥ 400 kcal) and a side dish; and (4) staple food without soup (≥ 400 kcal) and a side dish. The categories of dishes and combinations were based on the Japanese Food Guide Spinning Top and previous reports (14, 17). To further improve the accuracy of the dataset, two registered dietitians from external institutions identified inappropriate combinations—for example, a combination of significantly different dish styles and duplication of the dish category. The exclusion criterion was that at least one of the two dietitians judged a meal to be inappropriate. After excluding 208 inappropriate meals, we selected 792 meals for analysis. The nutritional values were calculated using Excel Eiyo-kun ver. 8, based on the Standard Tables of Food Composition in Japan (8th Revised Edition). The values were determined by calculating the energy and nutrient content of each dish based on the weight of its ingredients.

#### 2.3.3 Bento-box meals and restaurant meals

The take-away bento box and restaurant meals were selected using data from Foodbrowser^®^ (IMD Inc., Tokyo Japan). We selected data from “set meals/combos” and “bento/donburi” categories in order of the most recent update. From the extracted data, meals that were part of “meals/combos” and those intended for children were excluded from the analysis. Finally, 474 restaurant meals (some including breakfast meals) and 240 take-away bento-box meals were included in the analysis. The nutritional values were calculated based on the rules provide in the Foodbrowser^®^. Specifically, energy, protein, fat, carbohydrates, and sodium were used according to the content values published by the manufacturer. For nutrients that were not published, trained registered dietitians estimated the food weight based on an examination of the actual product, the ingredient information, and the manufacturer’s website information and calculated the component content using the Standard Tables of Food Composition in Japan (8th Revised Edition).

### 2.4 Calculation using the mHEI-2015 and NRF9.3

The Healthy Eating Index-2015 (HEI-2015) and the Nutrient-Rich Food Index 9.3 (NRF9.3) are well-validated dietary scores and nutrient profiling systems. In a previous study, these were used to assess meal quality in a Japanese sample (33). However, the HEI-2015 requires a specific database converted from servings, cups, and grams in the imperial system (34, 35), and is limited in its application for the evaluation of Japanese diets and meals. Meanwhile, the metric Healthy Eating Index-2015 (mHEI-2015) can work without a specific database due to its use of metric system; it has shown a high degree of comparability with the HEI-2015 (34). Therefore, the convergent validity of the ANPS-Meal was assessed in comparison with the mHEI-2015 and the NRF9.3.

The mHEI-2015 is a 100-point scale index that evaluates adherence to the 2015–2020 Dietary Guidelines for Americans using metric units (34), with higher scores indicating higher diet quality. The mHEI-2015 includes nine adequacy components (total fruits, whole fruits, total vegetables, greens and beans, whole grains, dairy calcium, total protein foods, seafood, plant protein foods, and the ratio of polyunsaturated and monounsaturated fatty acids to SFAs) and four moderation components (refined grains, sodium, added sugars, and saturated fatty acids), with a higher component point indicating favorable intake. We calculated the total and component scores of the mHEI-2015 based on energy-adjusted values for each meal (i.e., content per 1,000 kcal of energy or percentage of energy), except for fatty acids. The scoring standards used for the mHEI-2015 are detailed in Supplementary Table 1. To calculate the gram equivalent, the amount of food was multiplied by the following factor: for fruits, vegetables, legumes, and nuts, we used a factor of 1, as indicated in the raw values; for refined grains and whole grains, we uniformly used the factors of rice (0.46) and buckwheat (0.48), which are frequently used.

The NRF 9.3 is a composite measure of nutrient density, calculated as the sum of the percentage of reference daily values (RDVs) for nine nutrients to encourage minus the sum of %RDVs for the three nutrients to limit (36). The percentage of RDV was calculated as the ratio of the nutrient content of individual meals to one-third of the RDV and capped at 100. Therefore, a higher total score of NRF9.3 also indicates higher diet quality (36). For the RDVs of protein, SFAs, and sodium, the same daily values (DVs) as those used in the ANPS-Meal were applied. For other nutrients, the U.S. Food and Drug Administration daily values and Dietary Reference Intakes for the Japanese (2020) were used (37). The RDVs used for the NRF calculations are listed in Supplementary Table 2.

### 2.5 Statistical analyses

The nutrient values, component points, and total scores by quartiles of the ANPS-Meal score are presented as median and interquartile ranges (IQRs) or means with standard deviations (SDs). Linear models were used to examine the differences in the component points and nutrient values according to ANPS-Meal quartiles. Owing to zero counts in the lowest quartile (Q1) of the number of nutritionally recommended meals, we used Fisher’s exact test to assess the differences in the number of meals in each group by quartile. To test convergent validity, the associations between the ANPS-Meal and the mHEI-2015, as well as those between the ANPS-Meal and NRF9.3, were assessed using Spearman correlation coefficients. All statistical analyses were performed using R software version 4.1.3.

## 3 Results

### 3.1 Development of the ANPS-Meal

We selected the components of the ANPS-Meal for evaluation. The components and daily values of the ANPS-Meal were determined according to the ANPS model for dishes (14). NPMs usually include nutrients that are limited and related of public health concern (14). Thus, they can help assessing the implementation of dietary guidelines in Japan (17). Based on the Japanese Food Guide Spinning Top and previous studies (17, 38), a typical Japanese meal consists of a staple food, a main dish, soup, and side dishes. The staple food primarily provides a source of carbohydrates, whereas side dishes and soup supply vitamins, minerals, and dietary fiber. The main dish is served as a source of protein, fat, energy, and iron. Utilizing both dietary reference intake data (37) and findings from the National Health and Nutrition Survey (39) for adults aged 20–60 years, and considering the typical style of Japanese meals, the following food group and three nutrients were selected for the ANPS-Meal prototype: protein and vegetables as encouraged intake components and SFAs and sodium as limited intake components.

The daily values (DVs) for each nutrient and food group were determined based on a previously reported study (14). Briefly, the DVs for each component were set as follows: The DV for protein was set at 66 g per day, calculated from 1.1 g/kg/day for the average body weight of a 60-kg adult (40–44). The DV for vegetables was set at 350 g, as recommended by the Healthy Japan 21 project (45). The DV for SFAs was set at 22.2 g, derived from 10% of the total daily energy intake of the 2,000 kcal/day diet (46). The DV for sodium was set at 2,756 mg (equivalent to 7 g of salt), which is the mean value of the Japanese target (37). The equivalent salt content was calculated by multiplying the sodium content by 2.54.

Table 1 summarizes the scoring standards for the ANPS-Meal. The target values of the nutrient points for meals were based on the nutritional targets of one-third of the DVs. The total ANPS-Meal score was calculated from the nutrient points and converted into a 100-point scale as the following equation:


Total score of the ANPS−Meal=(PointsA+B+C+D)×2.5

**Table 1:** Scoring table of the ANPS-Meal.

Points	A	B	C	Points	D
	Protein	Vegetable	Saturated Fatty Acids		Sodium
	(g)	(g)	(g)		(mg)
10	≥22.0	≥116.7	≤7.41	10	≤917
9	≥19.8	≥105.0	≤8.15	9.5	≤1,009
8	≥17.6	≥93.4	≤8.89	9.0	≤1,101
7	≥15.4	≥81.7	≤9.63	8.5	≤1,193
6	≥13.2	≥70.0	≤10.37	8.0	≤1,284
5	≥11.0	≥58.4	≤11.12	7.5	≤1,376
4	≥8.8	≥46.7	≤11.86	7.0	≤1,468
3	≥6.6	≥35.0	≤12.60	6.5	≤1,559
2	≥4.4	≥23.3	≤13.34	6.0	≤1,651
1	≥2.2	≥11.7	≤14.08	5.5	≤1,743
0	<2.2	<11.7	>14.08	5.0	≤1,835
				4.5	≤1,926
				4.0	≤2,018
				3.5	≤2,110
				3.0	≤2,202
				2.5	≤2,293
				2.0	≤2,385
				1.5	≤2,477
				1.0	≤2,569
				0.5	≤2,660
				0.0	>2,660

### 3.2 Application of the ANPS-Meal to the evaluation for meals comsumed in Japan

**Figure 1:**
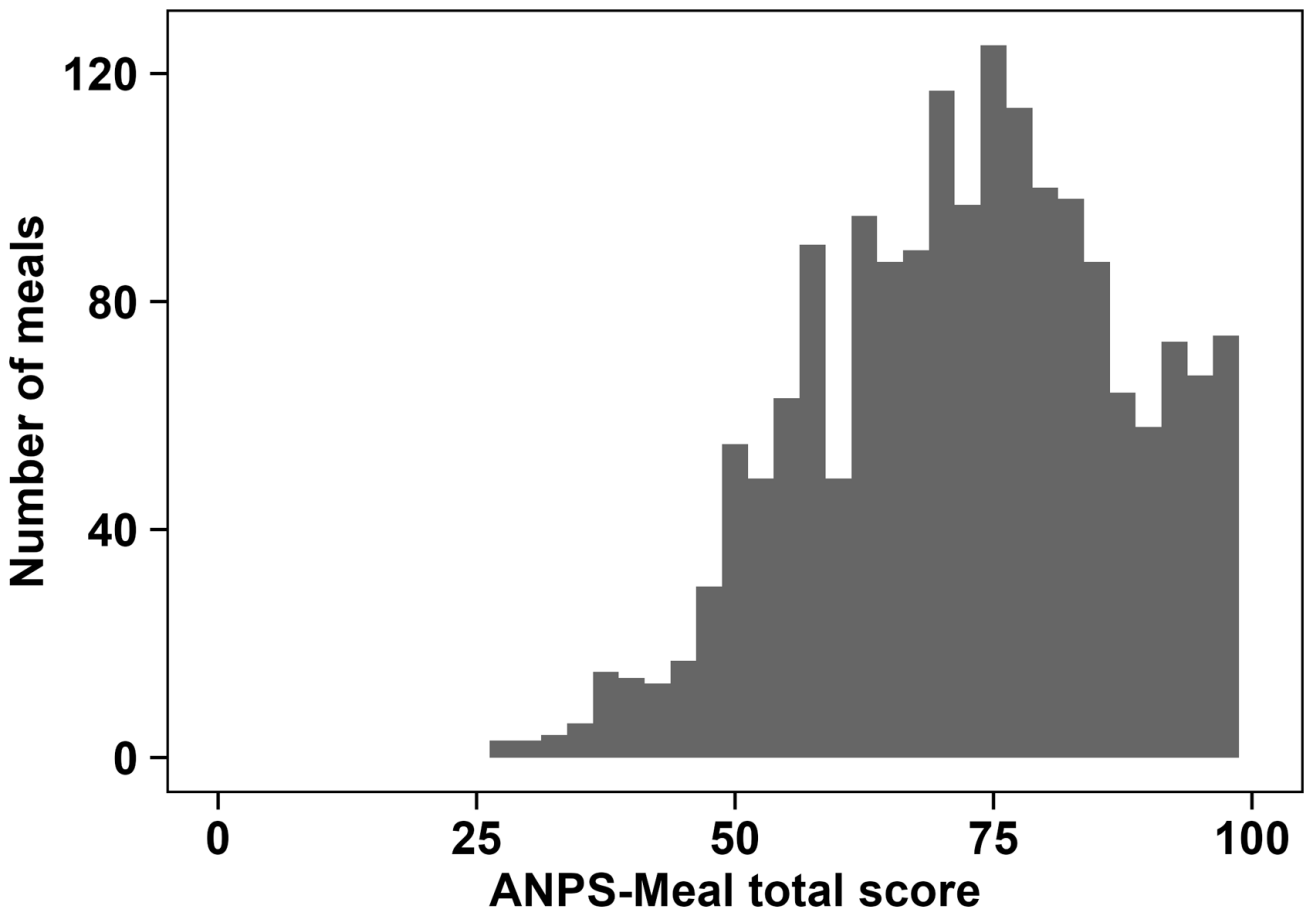
Distribution of the total meals score (*n* = 1,816 meals) evaluated by the ANPS-Meal. This shows the number of meals per 5-point increment in the ANPS-Meal.

Meals consumed in Japan were evaluated using the ANPS-Meal. The meals included combination of traditional Japanese, Western, and Chinese dishes, which are eaten in Japan. The distribution of the total ANPS-Meal scores is shown in Figure 1. The average total ANPS-Meal score was 73.7 (SD = 15.5). The median total ANPS-Meal score was 75 (IQR 62.5–85). The score distribution in the ANPS-Meal ranged from 27.5 to 100. The highest-scoring meals included low-sodium ingredients with high vegetables. The 56 nutritionally recommended meals and four randomly recommended meals were scored with 100 points. In contrast, the lowest-scoring meals contained high-sodium and high-SFAs ingredients with low vegetables. For example, chicken nanban (large size) with rice (fried chicken topped with a sweet vinegar sauce and served with tartar sauce and rice) and steak mix set meal (a meal with beef and chicken steak, fried prawns, spaghetti, and French fries) in restaurant meals and bento-box meals groups scored lower points (27.5 and 30). Table 2 summarizes the nutrient points of each component by quartiles of ANPS-Meal and the number of meals in each group by quartiles of the total score of ANPS-Meal. Higher quartiles were associated with higher vegetable points and lower SFAs and sodium points. In the highest quartile (Q4), vegetable points were the highest (mean 9.52, SD 1.03), whereas saturated fatty acids and sodium points were the lowest (saturated fatty acids points: mean 9.85, SD 0.58; sodium points: mean 8.56, SD 1.62). Nutritionally recommended meals were most frequently included in Q4. The randomly generated meals were equally distributed in Q2 and Q3, comprising 33 and 32% of the group, respectively. Bento-box meals were most frequently included in Q2, whereas restaurant meals were most frequently included in Q1. The energy and nutrient contents by quartile are listed in Table 3. For nutrient content, a higher ANPS-Meal score was associated with higher vegetable and lower sodium and saturated fatty acids. There was a negative association between energy intake and the total ANPS-Meal score. In terms of nutrient content, the higher quartiles of the ANPS-Meal were also associated with higher contents of carbohydrates, dietary fiber, potassium, calcium, magnesium, iron, vitamin A, vitamin D, and vitamin C, as well as lower contents of fat and sodium. However, there was no change in the mean value of the protein points according to quartiles of the total ANPS-Meal scores.

**Table 2:** Component points and number of meals in each group by quartiles of the ANPS-Meal total score^a^.

ANPS-Meal	Q1 (*n* = 468)					Q2 (*n* = 509)					Q3 (*n* = 411)					Q4 (*n* = 428)					*p* value
	Mean (SD)	Median	IQR			Mean (SD)	Median	IQR			Mean (SD)	Median	IQR			Mean (SD)	Median	IQR			
ANPS-Meal total score range	27.50–62.5					63.75–75.00					76.25–85.00					86.25–100					
Component points																					
Protein point^b^	9.5 (1.2)	10.0	10.0	–	10.0	9.5 (1.0)	10.0	9.0	–	10.0	9.4 (1.1)	10.0	9.0	–	10.0	9.5 (1.0)	10.0	10.0	–	10.0	0.588
Vegetable point^b^	3.8 (3.1)	3.0	1.0	–	6.0	4.8 (3.4)	4.0	2.0	–	8.0	7.4 (2.5)	8.0	5.0	–	10.0	9.5 (1.0)	10.0	10.0	–	10.0	<0.001
SFAs point^b^	4.9 (4.1)	5.0	0.0	–	10.0	8.7 (2.3)	10.0	8.0	–	10.0	9.4 (1.4)	10.0	10.0	–	10.0	9.8 (0.6)	10.0	10.0	–	10.0	<0.001
Sodium point^b^	3.2 (2.9)	3.0	0.0	–	5.5	5.0 (2.9)	5.0	3.0	–	7.0	6.1 (2.6)	6.0	4.0	–	8.0	8.6 (1.6)	9.0	7.5	–	10.0	<0.001
Meal group^c^																					
Nutritionally recommended meals	0% (0)					2% (6)					13% (40)					85% (264)					<0.001
Randomly generated meals	18% (140)					33% (261)					32% (257)					17% (134)					
Bento box meals	33% (80)					43% (102)					22% (52)					3% (6)					
Restaurant meals	52% (248)					30% (140)					13% (62)					5% (24)					

Q, quartile.

a The values are shown as mean, standard deviation (SD), median, and interquartile range (IQR).

b A linear trend test was applied with the median values in each quartile category of the total score of the ANPS-Meal (55, 70, 80 and 93.75, respectively) as continuous variables in a general linear regression model.

c Fisher’s exact test was applied.

**Table 3:** Associations of nutrient content and total score of the ANPS-Meal in 1,816 meals^a^.

Variables	Q1 (*n* = 468)			Q2 (*n* = 509)			Q3 (*n* = 411)			Q4 (*n* = 428)			*p* trend^b^
	Mean (SD)	Median	IQR	Mean (SD)	Median	IQR	Mean (SD)	Median	IQR	Mean (SD)	Median	IQR	
Energy (kcal)	846 (266)	828	643–1,016	686 (179)	666	543–800	631 (152)	609	522–715	563 (108)	551	496–608	<0.001
Protein (% energy)	15.9 (4.2)	15.7	13.1–18.1	17.2 (4.8)	16.5	13.6–20.3	17.7 (4.6)	17.3	14.2–20.8	19.0 (4.5)	18.5	16.0–21.2	<0.001
Fat (% Energy)	38.2 (12.6)	39.6	30.2–46.0	30.4 (10.5)	30.2	22.9–36.8	29.4 (9.8)	29.4	22.2–35.9	29.3 (8.2)	29.4	23.5–34.5	<0.001
Saturated Fatty Acids (% energy)	11.47 (5.69)	11.14	7.59–15.05	7.51 (3.92)	6.68	4.38–10.08	6.98 (3.46)	6.47	4.34–8.84	6.68 (2.89)	6.47	4.49–8.29	<0.001
Carbohydrate (% energy)	47.3 (12.1)	46.0	39.3–54.4	54.6 (10.2)	54.6	48.1–61.3	55.8 (9.8)	55.3	49.1–62.4	56.0 (8.6)	56.4	50.5–61.8	<0.001
Total Fiber (g/1,000 kcal)	6.9 (5.4)	5.2	3.1–9.4	10.1 (6.1)	9.0	5.5–13.4	11.8 (5.5)	11.2	8.1–15.6	14.6 (5.3)	13.7	11.0–17.5	<0.001
Sodium (mg/1,000 kcal)	2,878 (1169)	2,652	2,033–3,465	2,781 (1144)	2,577	1,871–3,522	2,567 (908)	2,573	1,814–3,260	1,947 (599)	1860	1,508–2,330	<0.001
Potassium (mg/1,000 kcal)	977 (405)	905	712–1,134	1,209 (573)	1,080	795–1,470	1,425 (559)	1,365	1,020–1,735	1,798 (532)	1736	1,377–2,174	<0.001
Calcium (mg/1,000 kcal)	173 (142)	127	82–215	189 (127)	161	99–242	226 (147)	196	128–279	281 (175)	238	160–359	<0.001
Magnesium (mg/1,000 kcal)	112 (51)	100	77–133	140 (62)	125	91–182	152 (61)	143	105–185	177 (57)	170	138–212	<0.001
Iron (mg/1,000 kcal)	3.7 (1.5)	3.6	2.7–4.5	4.3 (1.8)	3.9	2.9–5.4	4.6 (1.8)	4.4	3.2–5.5	5.4 (2.4)	5.1	3.7–6.5	<0.001
Vitamin A (μgRAE/1,000 kcal)	243 (864)	139	64–234	280 (770)	175	91–308	311 (321)	240	126–392	512 (1636)	347	187–542	<0.001
Vitamin D (μg/1,000 kcal)	3.3 (6.7)	1.0	0.6–3.1	4.8 (9.4)	1.4	0.5–4.4	4.2 (8.6)	1.1	0.4–4.4	5.5 (10.7)	1.3	0.4–5.3	<0.001
Vitamin C (mg/1,000 kcal)	22.9 (19.5)	18.0	10.2–29.9	29.7 (24.3)	22.4	12.7–41.0	44.8 (38.5)	36.4	22.5–56.5	92.4 (62.6)	79.2	48.2–122.0	<0.001
Vegetable (g/1,000 kcal)	65.5 (65.0)	49.8	28.7–79.0	104.7 (90.1)	81.7	43.6–136.1	182.7 (113.7)	158.6	110.6–213.8	280.5 (98.2)	265.9	209.1–350.6	<0.001

Q, quartile; RAE, retinol activity equivalent.

a The values are shown as mean, standard deviation (SD), median, and interquartile range (IQR).

b A linear trend test was applied with the median values in each quartile category of the total score of the ANPS-Meal (55, 70, 80 and 93.75, respectively) as continuous variables in a general linear regression model.

### 3.3 Testing the convergent validity of the ANPS-Meal

**Figure 2:**
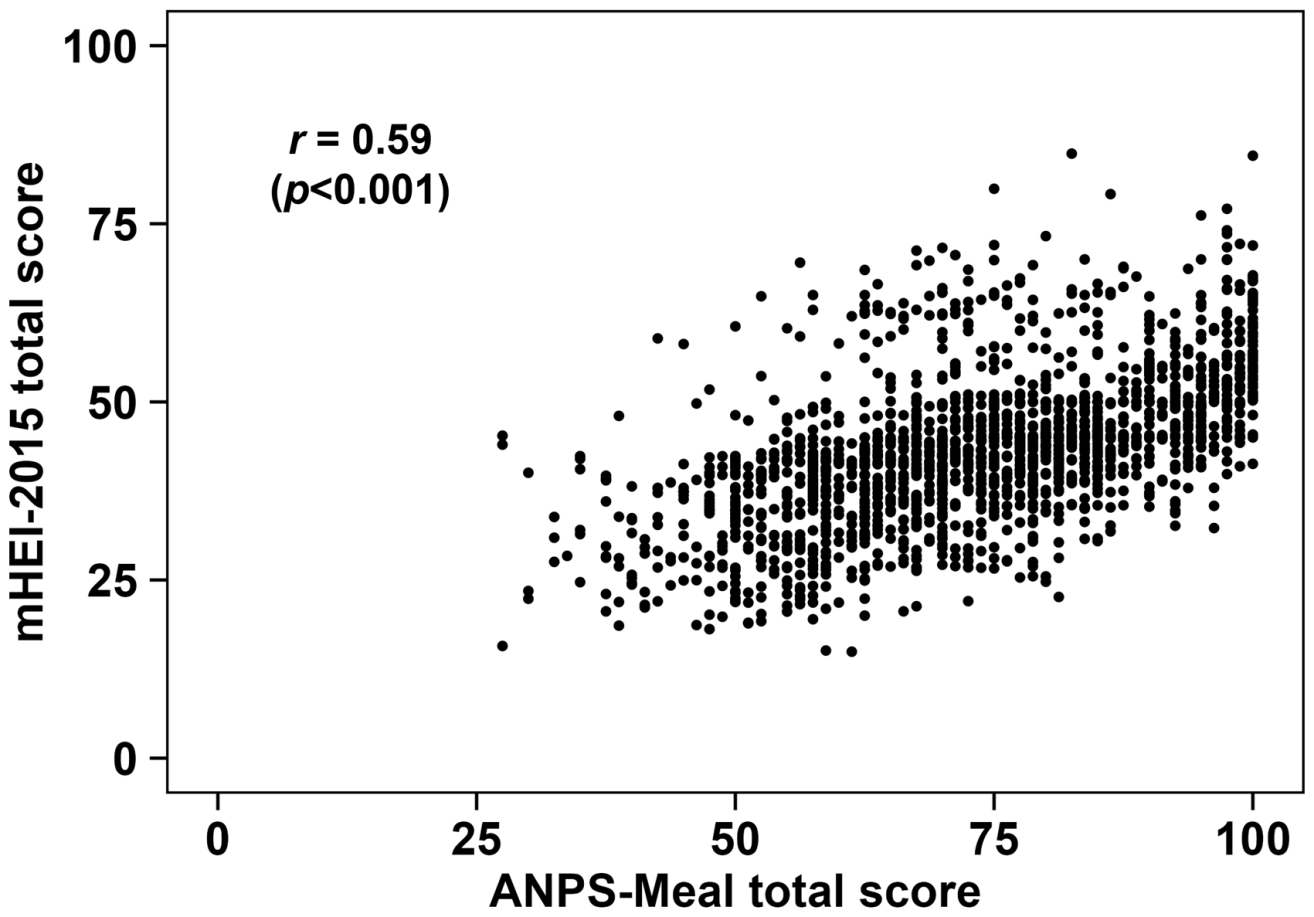
Scatter plot between the ANPS-Meal and the mHEI-2015. The Spearman correlation coefficient between the ANPS-Meal and the mHEI-2015 (*p* < 0.001) is shown.

**Figure 3:**
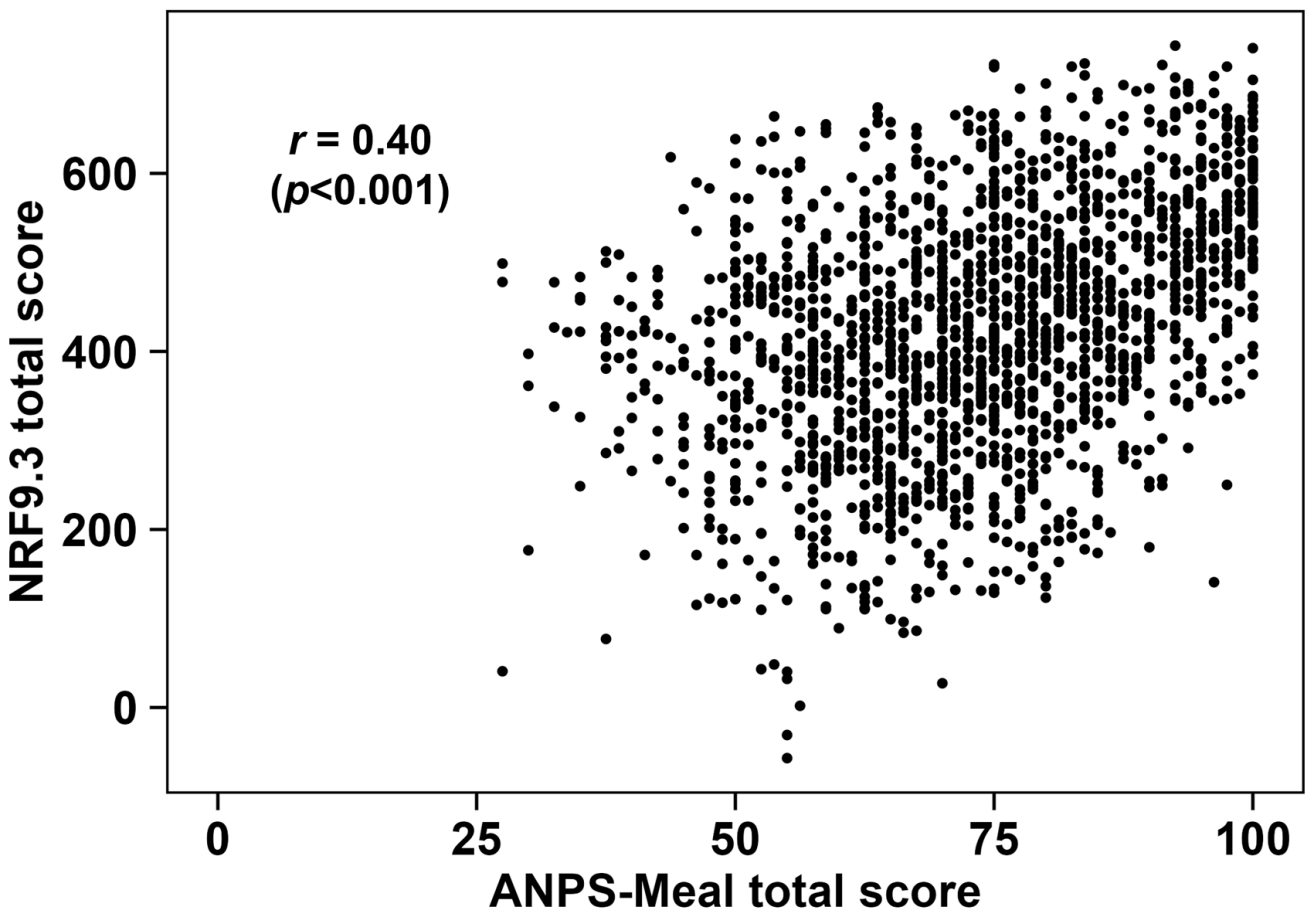
Scatter plot between the ANPS-Meal and the NRF9.3. The Spearman correlation coefficient between the ANPS-Meal and the NRF9.3 (*p* < 0.001) is shown.

Figure 2 shows a scatter plot of the ANPS-Meal compared with the mHEI-2015. Spearman correlation coefficients between the total score of the mHEI-2015 and the ANPS-Meal in 1,816 meals was *r* = 0.59 (*p* < 0.001). Figure 3 shows a scatter plot of the ANPS-Meal compared with the NRF9.3. Spearman correlation coefficients between the total score of the NRF9.3 and the ANPS-Meal in 1,816 meals was *r* = 0.40 (*p* < 0.001).

## 4 Discussion

The ANPS-Dish is a suitable NPM for evaluating the dishes consumed in Japan. In this study, we expanded the ANPS to the ANPS-Meal. To our knowledge, this is the first report of meal-based NPM to address public health issues specific to the Japanese population and evaluate meals consumed in Japan.

Total ANPS-Meal scores ranged from 27.5 to 100 points. Upon dividing the scores into quartiles, the upper-quartile group showed higher contents of energy-adjusted protein, carbohydrates, dietary fiber, potassium, calcium, magnesium, iron, vitamins A, D, and C, and vegetables, as well as lower contents of energy, fat, SFAs, and sodium. A closer look at the breakdown of the components of the ANPS-Meal revealed that the highest-quartile group had elevated points for vegetables, SFAs, and sodium. The protein component points were consistently high across all quartiles, with no significant differences. This may be attributed to the fact that more than half of the evaluated meals had a maximum score of 10. However, the range of ANPS-Meal total scores was sufficiently extensive, and a higher total score correlated with better nutritional quality of the entire meal (Table 3). The highest-quartile group predominantly included meals recommended by registered dietitians with strict control over the use of cooking oils, high-fat meat, and seasonings. The average energy content of the highest quartile was 562.6 kcal/meal, thus meeting the minimum standard value of 450 kcal/meal recommended by the Ministry of Health, Labour and Welfare for meals, aimed at preventing lifestyle-related diseases and promoting health (47). Meals planned by registered dietitians and traditional Japanese meals often combine main dishes and side dishes rich in vegetables, which are major sources of dietary fiber, potassium, and vitamin C. Meanwhile, the lowest-quartile group, with the lowest ANPS-Meal total score, had low energy-adjusted nutrient content for all nutrients except fat, SFAs, and sodium, and mainly consisted of restaurants and take-away bento-box meals. This is generally consistent with the findings of a previous study that indicated that eating out or eating takeout meals is associated with a deficiency in dietary fiber, vitamin C, and several minerals, as well as low vegetable intake (48). These results suggest that the ANPS-Meal has the potential to evaluate the overall nutritional value of various meals based on four dietary components.

The Spearman correlation coefficients between the ANPS-Meal and the mHEI-2015 and NRF9.3 were *r* = 0.59 and *r* = 0.40, respectively. Previous studies have reported correlation coefficients between NP systems applicable to meal evaluation in the range of *r* = 0.26–0.67 (49). Additionally, the correlation coefficient between total HEI-2015 scores and total NRF9.3 scores for breakfast, based on the US population, was *r* = 0.43 (50). Taken together, the correlation between ANPS-Meal scores and mHEI-2015 and NRF9.3 scores is generally consistent with previous studies, which suggests that the ANPS-Meal is useful in evaluating meals’ nutritional quality. The differences in these correlation coefficients may be due to the different assessment items used to calculate the scores. The ANPS-Meal and mHEI-2015 utilize the total amounts of specific food ingredients and nutrient content, while the NRF9.3 uses solely nutrient content. Furthermore, considering the ratio of qualifying nutrients to disqualifying nutrients in the NRF9.3, it is possible that the total score of NRF9.3 is less susceptible to disqualifying nutrients (added sugars, SFA, and sodium). Indeed, when examining nutrient contents by the quartile of each total score, the ANPS-Meal and mHEI-2015 exhibited similar trends, whereas the NRF9.3 showed no changes for sodium (Supplementary Tables 3, 4). Although it is important to consider that the NPM was developed for different purposes based on specific nutritional issues and food cultures, the differences in correlation between the ANPS-Meal, mHEI-2015, and NRF9.3 may be due to the reasons mentioned above.

This study has some limitations. First, as noted in a previous study, the novel ANPS-Meal is dependent on local food culture (14). Public health issues vary by country and region, and the WHO has emphasized the need for NP criteria to be developed in relation to the public health nutrition issues, culture, and environmental conditions of each country (1). Therefore, it is essential to develop an NPS that addresses the food culture and nutritional issues of each region and country. In fact, there are several movements in Japan considering their own unique NP (51–54). Second, because this study partially used randomly generated meals, the results may not fully reflect the diets consumed by Japanese people. Although dietitians excluded meals that could not realistically be eaten, it is assumed that there is considerable variation in meal combinations depending on the region, living arrangements, and household income, even within Japan (55–58). Therefore, the usefulness and accuracy of the ANPS-Meal must be verified. Finally, this study verified only the convergent validity of the ANPS-Meal. It is necessary to examine the relationship between the ANPS-Meal and health outcomes, as well as its predictive validity for disease onset. In the future, we aim to use this system as a tool for communicating with consumers and recommending meals to help them choose a nutritionally well-balanced diet.

## 5 Conclusion

The novel ANPS-Meal was developed to evaluate the nutritional value of meals consumed in Japan. The ANPS-Meal has the potential to comprehensively assess the overall nutritional value of various meals predominantly consumed in Japan, based on four components. Further research is required to determine the accuracy and utility of this model.

## Data Availability

The datasets presented in the article are not readily available because they are obtained from a third party source, Excel Eiyo-kun ver.8 (Kenpakusha,Tokyo, Japan) and Foodbrowser® (IMD Inc, Tokyo Japan). Requests to access these datasets should be directed to the corresponding author, Chie Furuta.
