# Supplementary material for: Development of the Ajinomoto Group Nutrient Profiling System for Japanese Meals
Source: Front Nutr. 2025 May 21;12:1568181. doi: 10.3389/fnut.2025.1568181 (PMC12133537; doi:10.3389/fnut.2025.1568181)
Supplement: Supplementary file 1 [file Table_1.docx]

Supplementary Material

## Supplementary Table 1. Scoring standards for the metric Healthy Eating Index-2015

| Component | Maximum score | Standard value for maximum score ^1^ | Standard value for minimum score ^1^ |
| --- | --- | --- | --- |
| **Adequacy** |  |  |  |
| Total fruits | 5 | ≥ 141 g equivalents | No total fruits |
| Whole fruits | 5 | ≥ 60 g equivalents | No whole fruits |
| Total vegetables | 5 | ≥ 160 g equivalents | No total vegetables |
| Greens and beans | 5 | ≥ 29 g equivalents | No greens and beans |
| Whole grains | 10 | ≥ 31 g equivalents | No whole grains |
| Dairy | 10 | ≥ 412 mg calcium | No dairy |
| Total protein foods | 5 | ≥ 15.6 g protein | No total protein |
| Seafood and plant proteins | 5 | ≥ 3.3 g protein | No seafood and plant proteins |
| Fatty acids | 10 | (PUFAs+MUFAs)/SFAs ≥ 2.5 | (PUFAs+MUFAs)/SFAs ≤ 1.2 |
| **Moderation** |  |  |  |
| Refined grains | 10 | ≤ 32 g equivalents | ≥ 76 g equivalents |
| Sodium | 10 | ≤ 1.1 g | ≥ 2.0 g |
| Added sugars | 10 | ≤ 6.5 % of energy | ≥ 26 % of energy |
| Saturated fatty acids | 10 | ≤ 8 % of energy | ≥ 16 % of energy |

PUFAs, polyunsaturated fatty acids. MUFAs, monounsaturated fatty acids. SFAs, saturated fatty acids

1 per 1,000kcal

**Supplementary Table 2.** Reference daily values for the NRF9.3

| Nutrient | Amount |
| --- | --- |
| Protein | 66 g ^1^ |
| Fiber | 21 g ^2^ |
| VitaminA | 900 μgRAE ^2^ |
| VitaminC | 100 mg ^2^ |
| Ca | 800 mg ^2^ |
| Fe | 11 mg ^2^ |
| Potassium | 3,000 mg ^2^ |
| VitaminD | 8.5 μg ^2^ |
| Mg | 370 mg ^2^ |
| AddedSugars | 50 g ^3^ |
| SFAs | 22.2 g ^1^ |
| Sodium | 2,756 mg ^1^ |

RAE, retinol activity equivalent.

1 Adapted from daily values of the ANPS.

2 Adapted from daily values of the Dietary Reference Intakes for Japanese (2020).

3 Adapted from the Food and Drug Administration value.

## Supplementary Table 3. Associations of nutrient content and total score of the mHEI-2015 in 1,816 meals ^1^

|  | **Q1 (n = 468)** | | | **Q2 (n = 509)** | | | **Q3 (n = 411)** | | | **Q4 (n = 428)** | | | *P*  trend ^2^ |
| --- | --- | --- | --- | --- | --- | --- | --- | --- | --- | --- | --- | --- | --- |
|  | **Mean (SD)** | **Median** | **IQR** | **Mean (SD)** | **Median** | **IQR** | **Mean (SD)** | **Median** | **IQR** | **Mean (SD)** | **Median** | **IQR** |  |
| Energy  (kcal) | 753 (234) | 724 | 581–881 | 738 (220) | 692 | 566–881 | 661 (193) | 616 | 537–743 | 591 (162) | 553 | 491–648 | <0.001 |
| Protein   (% Energy) | 16.0 (4.2) | 15.4 | 13.2–18.0 | 16.8 (4.9) | 16.1 | 13.5–19.4 | 18  (5) | 17.5 | 14.5–20.8 | 19.1 (4.4) | 18.7 | 16.2–21.3 | <0.001 |
| Fat  (% Energy) | 36.5 (11.7) | 36.8 | 28.7–44.4 | 32.1 (11.5) | 32.2 | 24.7–39.7 | 30  (9) | 29.8 | 22.5–35.4 | 29.4 (10.1) | 28.2 | 22.2–35.6 | <0.001 |
| Saturated fatty acids  (% Energy) | 12.40 (4.82) | 12.30 | 9.47–15.07 | 8.03 (4.17) | 7.70 | 4.99–10.19 | 6.30 (3.11) | 5.70 | 3.98–8.07 | 6.14 (2.94) | 5.70 | 4.02–7.71 | <0.001 |
| Carbohydrate  (% Energy) | 49.6 (11.9) | 48.7 | 40.9–57.6 | 53.2 (11.2) | 53.1 | 46.0–60.9 | 55.4 (9.2) | 55.4 | 49.8–62.0 | 55.1 (10.1) | 55.8 | 49.3–61.8 | <0.001 |
| Total Fiber  (g/1,000kcal) | 8.1 (5.6) | 6.7 | 3.6–10.9 | 9.1 (5.8) | 8.2 | 4.2–12.5 | 11.5 (6.1) | 10.7 | 7.7–14.7 | 14.2 (5.7) | 13.9 | 10.6–17.2 | <0.001 |
| Sodium  (mg/1,000kcal) | 2,715 (1003) | 2,555 | 2,037–3,226 | 2,692 (1,049) | 2,470 | 1,889–3,284 | 2,554 (1,045) | 2,335 | 1,772–3,121 | 2,283 (1,076) | 1,941 | 1,459–3,003 | <0.001 |
| Potassium  (mg/1,000kcal) | 1,059 (468) | 964 | 734–1,245 | 1,175 (511) | 1,064 | 813–1,452 | 1,404 (592) | 1,308 | 972–1,734 | 1,710 (606) | 1,710 | 1,237–2,134 | <0.001 |
| Calcium  (mg/1,000kcal) | 165 (130) | 124 | 77–204 | 181 (135) | 147 | 98–219 | 224 (145) | 194 | 129–278 | 290 (170) | 248 | 169–374 | <0.001 |
| Magnesium  (mg/1,000kcal) | 107 (41) | 99 | 79–124 | 126 (48) | 118 | 90–153 | 152 (58) | 146 | 108–189 | 193 (63) | 190 | 149–229 | <0.001 |
| Iron  (mg/1,000kcal) | 3.8 (1.4) | 3.7 | 2.8–4.7 | 3.8 (1.7) | 3.60 | 2.7–4.7 | 4.3  (2) | 4.1 | 3.0–5.3 | 5.8 (2.3) | 5.6 | 4.4–7.0 | <0.001 |
| Vitamin A  (μgRAE  /1,000kcal) | 226 (210) | 177 | 83–297 | 345 (1,333) | 169 | 83–301 | 337 (884) | 217 | 107–368 | 419 (1,214) | 302 | 153–511 | 0.005 |
| Vitamin D  (μg/1,000kcal) | 1.9 (2.9) | 0.9 | 0.5–2.5 | 3.6 (7.7) | 1.1 | 0.3–3.1 | 5  (10) | 1.4 | 0.4–5.1 | 7.1 (12.2) | 2.2 | 0.6–6.4 | <0.001 |
| Vitamin C  (mg/1,000kcal) | 29.7 (24.9) | 22.1 | 13.4–37.4 | 33.5 (30.5) | 23.9 | 12.7–43.6 | 45 (41) | 34.0 | 18.3–57.4 | 76.2 (66.4) | 57.3 | 27.6–98.5 | <0.001 |
| Vegetable  (g/1,000kcal) | 106.1 (94.8) | 74.1 | 39.4–144.5 | 123.6 (109.6) | 89.4 | 43.3–171.3 | 164 (121) | 143.1 | 70.1–230.7 | 221.0 (131.9) | 209.3 | 116.9–305.4 | <0.001 |

Q, quartile. RAE, retinol activity equivalent.

1 The values were shown as mean, standard deviation (SD), median and inter-quartile range (IQR).

2 A linear trend test was applied with the median values in each quartile category of the total score of the mHEI-2015 (31.5, 40.9, 45.2 and 54.8, respectively) as continuous variables in a general linear regression model.

## Supplementary Table 4. Associations of nutrient content and total score of the NRF9.3 in 1,816 meals ^1^

|  | **Q1 (n = 468)** | | | **Q2 (n = 509)** | | | **Q3 (n = 411)** | | | **Q4 (n = 428)** | | | *P*  trend ^2^ |
| --- | --- | --- | --- | --- | --- | --- | --- | --- | --- | --- | --- | --- | --- |
|  | **Mean (SD)** | **Median** | **IQR** | **Mean (SD)** | **Median** | **IQR** | **Mean (SD)** | **Median** | **IQR** | **Mean (SD)** | **Median** | **IQR** |  |
| Energy  (kcal) | 694 (206) | 659 | 536–837 | 700 (223) | 653 | 531–810 | 694 (229) | 634 | 542–774 | 655 (195) | 591 | 521–747 | 0.007 |
| Protein   (% Energy) | 14.8 (4.3) | 14.3 | 12.2–16.7 | 17.0 (4.1) | 16.4 | 14.1–19.6 | 19  (5) | 18.0 | 15.5–21.3 | 19.2 (4.4) | 18.7 | 16.3–21.6 | <0.001 |
| Fat  (% Energy) | 33.2 (11.8) | 32.9 | 25.5–41.0 | 31.6 (11.8) | 31.1 | 22.7–38.7 | 32  (11) | 31.4 | 25.0–38.0 | 31.0 (10.1) | 30.6 | 23.6–37.4 | 0.005 |
| Saturated fatty acids  (% Energy) | 9.12 (4.84) | 8.90 | 4.98–12.09 | 8.32 (4.90) | 7.50 | 4.46–10.97 | 7.95 (4.28) | 7.00 | 4.80–10.07 | 7.47 (4.15) | 6.50 | 4.52–9.29 | <0.001 |
| Carbohydrate  (% Energy) | 53.3 (11.8) | 53.7 | 45.4–61.1 | 53.5 (11.4) | 53.8 | 46.1–61.4 | 53  (10) | 53.1 | 45.9–59.6 | 53.8 (10.1) | 54.4 | 47.2–60.9 | 0.718 |
| Total Fiber  (g/1,000kcal) | 6.7 (4.6) | 5.4 | 3.1–9.3 | 9.4 (5.2) | 8.9 | 5.2–12.1 | 12  (6) | 11.9 | 8.0–15.7 | 14.9 (6.1) | 14.4 | 10.6–18.2 | <0.001 |
| Sodium  (mg/1,000kcal) | 2,403 (1,099) | 2,155 | 1,645–2,848 | 2,636 (1,031) | 2,444 | 1,849–3,232 | 2,709 (1,081) | 2,616 | 1,886–3,376 | 2,496 (989) | 2,330 | 1,719–3,083 | 0.094 |
| Potassium  (mg/1,000kcal) | 854 (341) | 788 | 635–997 | 1,150 (399) | 1084 | 883–1,354 | 1,471 (522) | 1,416 | 1,115–1,777 | 1,873 (569) | 1,826 | 1,458–2,271 | <0.001 |
| Calcium  (mg/1,000kcal) | 127 (95) | 97 | 63–162 | 175 (108) | 151 | 109–205 | 226 (132) | 199 | 141–280 | 331 (182) | 292 | 207–404 | <0.001 |
| Magnesium  (mg/1,000kcal) | 99 (37) | 89 | 73–117 | 128 (50) | 118 | 94–151 | 156 (54) | 153 | 113–191 | 194 (62) | 189 | 147–232 | <0.001 |
| Iron  (mg/1,000kcal) | 3.1 (1.2) | 2.9 | 2.2–3.8 | 4.0 (1.4) | 3.8 | 2.9–4.8 | 4.6 (1.4) | 4.6 | 3.5–5.5 | 6.1 (2.4) | 5.9 | 4.6–7.2 | <0.001 |
| Vitamin A  (μgRAE  /1,000kcal) | 134 (167) | 93 | 45–174 | 302 (1148) | 172 | 89–277 | 294 (318) | 228 | 142–347 | 598 (1592) | 429 | 278–626 | <0.001 |
| Vitamin D  (μg/1,000kcal) | 1.3 (2.3) | 0.6 | 0.2–1.2 | 3.9 (7.7) | 1.3 | 0.5–3.9 | 5.3 (11.1) | 1.4 | 0.5–4.2 | 7.2 (10.8) | 3.6 | 1.0–7.4 | <0.001 |
| Vitamin C  (mg/1,000kcal) | 20.0 (17.5) | 16.3 | 9.4–24.7 | 33.1 (31.9) | 24.6 | 13.5–40.5 | 48.5 (43.3) | 36.0 | 22.3–62.9 | 83.0 (59.7) | 63.2 | 42.3–103.9 | <0.001 |
| Vegetable  (g/1,000kcal) | 78.1 (81.4) | 56.7 | 30.7–103.3 | 119.0 (92.9) | 98.7 | 49.1–167.1 | 170.9 (117.7) | 146.9 | 79.5–234.6 | 246.7 (126.3) | 232.7 | 155.5–323.4 | <0.001 |

Q, quartile. RAE, retinol activity equivalent.

1 The values were shown as mean, standard deviation (SD), median and inter-quartile range (IQR).

2 A linear trend test was applied with the median values in each quartile category of the total score of the NRF9.3 (261.9, 385.8, 474.7 and 586.4, respectively) as continuous variables in a general linear regression model.
